# Tranquillizers

**Published:** 1962-01

**Authors:** R. E. Hemphill

**Affiliations:** Lecturer in Charge of the Department of Psychiatry, University of Bristol. Consultant Psychiatrist, United Bristol Hospitals


### TRANQUILLIZING AND ANTI-PSYCHOTIC DRUGS IN OUTPATIEN[illegible] AND GENERAL PRACTICE

BY

R. E. HEMPHILL, M.A., M.D., D.P.M.,

*Lecturer in Charge of the Department of Psychiatry, University of Bristol.*
*Consultant Psychiatrist, United Bristol Hospitals*

The last ten years have seen the introduction of many new drugs for the treatme[illegible] of psychotic disorders and practically every psychiatric symptom. They are alrea[illegible] replacing the barbiturates which have been used as sedatives for over 50 years.

These drugs aim at influencing the processes concerned in the development of psychiatric illness, e.g. schizophrenia and depression, or at controlling the mo[illegible] disturbing phenomena such as agitation, anxiety, or tension.

The sedatives, and particularly barbiturates, have a hypnotic and functional[illegible] depressing effect in proportion to their potency and their action is mainly on high[illegible] cerebral function. Many of the new drugs have no hypnotic effect. Some cause i[illegible] creased alertness and may produce extra-pyramidal disturbances and other eviden[illegible] of an action on the reticular system and deeper structures of the brain. It must [illegible] stated however, that in spite of the theoretical neuro-pharmacology, there is no co[illegible] clusive evidence about how they influence the production of mental symptoms, a[illegible] even less about how their action may be related to a neuro-physiological origin [illegible] psychosis. Even in the group of mono-amine-oxidase inhibitors, the theory th[illegible] psychosis is influenced by their effect upon certain enzyme systems in the brain [illegible] completely unproven and it has been suggested that this action is totally irrelevant, [illegible] indeed it takes place in humans.

Nevertheless, it is established that these drugs do relieve certain psychiatric illness[illegible] and it is possible, even at this early stage, to say what the indications are and how si[illegible] effects and undesirable complications can be minimized.

The first of these drugs to be introduced was Chlorpromazine which was call[illegible] rather erroneously a tranquillizer because it calmed agitation, restlessness, and psych[illegible] tic behaviour, but the name suggested that it produced mental tranquillity which [illegible] quite a different matter. This has been responsible for much of the inappropria[illegible] use of the Promazine drugs. Chlorpromazine had its early striking success in th[illegible] treatment of disturbed, hallucinated patients in the long-stay wards of large ment[illegible] hospitals and not in tranquillizing anxious individuals in the community.

The drugs can conveniently be divided into three groups: the so-called tranquillize[illegible] mainly useful for psychotic disturbances and agitation; the thymoleptics for depressio[illegible] and an indeterminate group partaking of both the tranquillizer and the thymolept[illegible] effect.

The effect of these drugs in psychotic patients is not necessarily the same as in no[illegible] psychotics. This should be borne in mind when prescribing. Failure to realize th[illegible] the response to powerful phenothiazines in normal individuals suffering from anxie[illegible] may be vastly different in extent and nature from similar treatment in schizophreni[illegible] has been responsible for alarming reactions and therapeutic failures.

The names of the majority of the preparations on the market, with comments o[illegible] dosage, side effects and indications, are given in the accompanying table. Some of th[illegible] earlier preparations have been largely superseded. No psychiatrist is likely to use a[illegible] these drugs and the favourable comments and observations are the result of person[illegible] experience but do not necessarily assume that some of the other preparations are not [illegible] equal value. Side effects and contra-indications refer to what has been published [illegible]

well as personal experience. The familiar trade names will be used in this paper for the sake of clarity; the pharmacological names are given in brackets in the table.

### TRANQUILLIZERS

| | *Daily Dose* | *Injection* | *Side Effects* | *Indications and Contra-indications* |
|---|---|---|---|---|
| PHENOTHIAZINE | | | | |
| LARGACTIL (M. & B.) (Chlorpromazine) | 75–1200 mgm | 50–100 mgm | Contact dermatitis Liver damage Parkm. Hypotension | Psychotic agitation Schizophrenia |
| STELAZINE (S.K.F.) (Trifluoperazine) | 2–30 mgm | 1–2 mgm | as above | as above |
| FENTAZIN (A. & H.) (Perphenazine) | 6–24 mgm | 5 mgm | as bove Parkm. + | as above |
| MAJEPTIL (M. & B.) (Thioproperazine) | (a) 300 mgm (b) 15 mgm | 7·5 mgm repeated | Neurological + Intestinal obstruction | Mania. Anti-emitic + |
| MELLERIL (Sandoz) (Thioridazine) | 75–600 mgm | — | — | Psycho-sedative for anxiety and tension No anti-emetic. No potentiating effects No jaundice |
| SPARINE (Wyeth) (Promazine) | 75–600 mgm | 50–100 mgm | Slight | Geriatrics. Seniles No liver |
| FRENQUEL (Ricker) (Azocyclonal) | 100 mgm | — | — | — |
| STEMETIL (M. & B.) (Prochloperazine) | 15–75 mgm | — | As Largactil | As Largactil |
| VERACTIL (M. & B.) (Methotrimeprazine) | 75–600 mgm | — | as above | as above |
| VESPRAL (Squibb) (Trifluopromazine) | 75–300 mgm | — | as above | as above |
| PACATAL (Warner) (Pecazine) | 75–300 mgm | — | as above | as above |
| NOTENSIL (Benger) (Acetylpromazine) | 75–150 mgm | — | as above | as above |
| MODITEN (Squibb) (Fluphenazine) | 1–2 mgm single dose a.m. | — | slight | Not to be used for severe depression or history of convulsions Mainly for anxiety tension |
| DARTALAN (Searle) (Thiopropazate) | 15–30 mgm | — | — | — |
| SERENACE (Searle) (Haloperidal) | 0·75–6 mgm | 5 mgm | — | — |
| EQUANIL (Wyett) (Meprobamate) | 600–1200 mgm | — | — | very mild |
| MILTOWN (Lederle) (Meprobamate) | 600–1200 mgm | — | — | very mild |
| MEPAVLON (I.C.I.) (Meprobamate) | 600-1200 mgm | — | — | very mild |

| | *Daily Dose* | *Injection* | *Side Effects* | *Indications and Contra-Indications* |
|---|---|---|---|---|
| OTHERS | | | | |
| LIBRIUM (Roche) (Chlordiazepoxide) | 20–100 mgm | — | — | Reduction of fear and aggression<br>Relief of tension and insomnia<br>Relief of muscle spasm in painful arthritis<br>No extra-pyram.<br>No autonomic blocking |
| TARACTAN (Roche) (Chlordiazepoxide) | 45–300 mgm | — | — | Tranquillizing and anti depressant<br>Calming epileptic type of behaviour<br>Rapid acting<br>Potentiates alcohol and narcotics |
| ANARGYL (M. & B.) | (Largactil 25 mgm<br>(Amylobarb. 50 mgm) | | | |
| AMYLOZINE (S.K.F.) | (Stelazine 2 mgm)<br>(Amylobarb. 60 mgm) | | | |
| SERPASIL (Ciba) (Reserpine) | 3–10 mgm | — | — | Extra-pyram. +<br>? causes depression |
| NITOMAN (Roche) (Tetrabenazine) | 75–300 mgm | — | — | as above |
| THYMOLEPTICS | | | | |
| *M. A. O. Inhibitors* | | | | |
| NARDIL (Warner) (Phenelzine) | 30–75 mgm | — | — | Claimed to be safe |
| NIAMID (Pfizer) (Malanide) | 75–300 mgm | — | — | — |
| PARNATE (S.K.F.) (Tranylcypromine) | 10–30 mgm | — | — | Depression. Dose early in the day. May cause insomnia. Non-toxic<br>No liver damage<br>Claimed effective in 20 hours |
| MARPLAN (Roche) (Isocarboxazid) | 10–30 mgm | — | — | Toxic. More potent than Marsilid |
| MARSILID (Roche) (Iproniazid) | 150 mgm | — | — | Toxic |
| CAVODIL (Bengers) (Pheniprazine) | 6–18 mgm | — | Liver damage<br>Hypotens. +<br>Blurred Vis.<br>Red/green defect<br>Skin React.<br>Oedema | As in Marplan<br>Used for angina pain |
| OTHER THYMOLEPTICS | | | | |
| TOFRANIL (Geigy) (Imipramine) | 50–250 mgm | 150 mgm | Dry mouth<br>Hopytens.<br>Parkm. | Powerful anti-depressant |
| TRIPTIZOL (M. S. & D.) (Amitriptyline) | 30–75 mgm | — | Slight | Endogenous depression |
| PARSTELIN (S.K.F.) | Parnate 10 mg.<br>(Stelazine 1 mg) | — | Slight<br>Insomnia | Anxiety attacks.<br>Anxiety depression<br>Reactive depression |

| | *Daily Dose* | *Injection* | *Side Effects* | *Indications and Contra-Indications* |
|---|---|---|---|---|
| ANTI-PARKINSON DRUGS | | | | |
| ARTANE (Lederle) (Benzhexol) | 4–10 mgm | — | — | — |
| PIPANOL (Bayer) (Benzhexol) | 4–10 mgm | — | — | — |
| COGETIN (M. S. & D.) (Benztropine) | 2–6 mgm | — | — | — |
| PHENERGAN (M. & B.) (Promethazine HCI) | 25–50 mgm | — | — | — |
| KEMADRIN (B. Ward & Co.) (Procyclidine) | 2·5–5 mgm | — | — | — |

*Doses*

The "Maximum dose" is the estimated permissible maximum dose in 24 hours under any circumstances. It is greatly in excess of what will normally be used outside hospital or if the patient is not under constant observation.

The doses and regime recommended by the makers can generally be followed but it is advisable to start with small doses in new cases. Side effects as well as clinical response should be considered in increasing the dose.

The most effective tranquillizers and thymoleptics are powerful drugs, and must be used with much greater care than the barbiturates and other sedatives. They should not be prescribed unless the doctor has a clear therapeutic aim in view, and reasons for his choice of drug and dosage.

The therapeutic aims are (*a*) treatment of the psychiatric disorder, and (*b*) relief of symptoms. Symptomatic improvement such as the relief of insomnia or anxiety is probably more important in psychiatry than in other branches of medicine, because until it is secured the patient may be unable to apply himself to the wider problems of his illness, will not have confidence, and may well try other forms of treatment as an alternative or an addition.

It is not permissible to give tranquillizers as placebos, and if reassurance only is required, some innocuous preparation or mild hypnotic should be prescribed. With some tranquillizers or thymoleptics, undersirable or dangerous side effects are produced, e.g. hypotensive attacks and extra-pyramidal Parkinsonism. These may be signs of overdosesage or incompatibility and in the majority of cases the patient should be treated in hospital if they persist. Continuing mental dulling, loss of initiative, and slowing may also be due to overdosage.

A main principle of therapy should be to arrive at a dose which controls the illness but avoids mental inertia, unpleasant side effects, addiction or dependence.

### ACUTE CONDITIONS

For settling a wild and excited patient before admission to hospital, most of the new drugs are unsatisfactory, both because their action is uncertain and slow and because of the risk of collapse due to hypotension, especially in exhaustion, cardio-vascular disease and old age. A single injection of the old morphine-hyoscine combination is still the best in these cases.

Excitement, restlessness and violent behaviour are usually due to one of the following:—acute mania, schizophrenia, alcohol, confusion, brain damage, epilepsy, and delirium due to toxemia or drugs. For these, intra-muscular Largactil (50—100 mgm),

Stelazine (1–2 mgm) or Serenace (5 mgm) repeated, may be effective but should not be given to the ambulant patient. For senile restlessness, Sparine (50–100 mgm) by injection will cover an emergency without much risk. These drugs may potentiate the action of barbiturates and alcohol, and must be used with caution if there is a suspicion that the patient has already been taking either. It should not be forgoten that acute excitment and restlessness may be due to cerebral tumour, head injury, or recent epileptic fit and that tranquillizers may mask the real diagnosis.

### OTHER PSYCHIATRIC CONDITIONS

### *Anxiety States*

Some tranquillizers help in reducing muscular tension and in controlling surges of fear and acute panic. They are not cures but are aids to psychotherapy. Unless the cause is elicited, the patient is liable to become dependent upon the drug without getting well. Since patients with anxiety are usually sensitive to the phenothiazines the initial dose should be low. Emotional and sympathetic lability may produce severe reactions with hypotensive drugs. If the patient complains of giddiness, sweating and blacking-out, increasing with treatment, the drug is probably responsible and it should be stopped or the dose reduced. A common mistake is to increase the dose in the belief that these effects are due to the neurosis. Unsteadiness and postural giddiness are important signs of overdosage or intolerance.

A barbiturate should not be given by day with tranquillizers and if used for insomnia the dose should be small. Some of the other hypnotics are preferable and Librium may be used as a night sedative or hypnotic.

If there is no improvement in a week or two the drug is not suitable. Otherwise a dose should be found that controls symptoms and after about another two weeks gradually reduced. One should not envisage the continuous treatment of anxiety with these drugs for more than three months.

For patients who develop anxiety before a stressful situation, such as appearing in public, a small dose of one of the tranquillizers, particularly Parstelin, may be useful but treatment should be limited to avoid dependency.

### *Obsessional States*

Apart from relieving anxiety or depression secondary to obssesions, these drugs are ineffective in obsessional neurosis.

### *Hysterical States*

There is no point in prescribing tranquillizers for hysterics and to do so tends to fix the idea of illness more firmly and obscure the real motivation.

### *States of Aggression and Tension*

There is no doubt that some aggressive patients and tense individuals under stress are calmed and feel subjectively better with some tranquillizers. They become more tolerant and this in turn helps their environment. Tension pains in the neck and back muscles, common in anxiety-tension and states of aggression and frustration may be relieved. Among the drugs indicated, Librium and Melleril are valuable and there is evidence that both can influence an abnormal E.E.G. and aggressive behaviour in epileptics.

*Mania*

Since most manics refuse to come into hospital they may have to be treated as outpatients, although this is undesirable. All the thymoleptic drugs are contra-indicated but the tranquillizers may be used. The powerful preparations Majeptil and Serenace are effective but it is preferable to use them only on inpatients.

*States of Depression*

States of depression include cyclothymic, periodic depression of endogenous type; reactive depression in which the illness is a reaction to some provoking event; and anxiety depression in which the depressive state is associated with anxiety.

The various thymoleptics influence depression and can terminate mild attacks in a proportion of cases.

It is doubtful whether more than 30 per cent of established cases of endogenous depression clear up with drugs alone. For severe depression, electro-shock is far safer because of the risk of suicide and, in general, unless the depressive attack has shown steady improvement within ten days drug treatment, E.C.T., should be considered. The principal disadvantage of E.C.T. is disturbamce of memory after more than four treatments and it is often convenient to start the case with about three electrical treatments, continuing with a thymoleptic drug.

There is a considerable choice of drugs. All of them have advocates. Tofranil is probably the most widely used for depression but Parstelin, a mixture of Parnate and Stelazine, appears to be particularly effective in anxiety depression.

One must envisage about three months' treatment with an anti-depressant drug, tailing off the dose gradually if relapse is to be avoided.

*Prophylatic treatment of Recurrent Depression*

The thymoleptics are particularly useful for continuous treatment of patients who have numerous depressive attacks with short intervals. Experience up to the present suggests that continuous treatment in low dosage may keep a patient free from attacks indefinitely.

In all cases of depression morbid ideas, reduction in activity, or failure to maintain the rate of improvement are ominous. It is worth stressing that depression is a dangerous condition because of the risk of suicide, disabling because of inability to work, and mentally painful to the patient. It is not permissible to defer electrical treatment too long if thymoleptic drugs are not being really effective.

*Schizophrenia*

The majority of cases of acute schizophrenia will be treated in hospital. The phenothiazines, including the original Largactil and the more powerful Stelazine, are satisfactory and widely used.

Many cases of schizophrenia, chronic or recent, can be treated as outpatients when a suitable drug and dosage have been arrived at. As these patients are bad witnesses it is important to look for signs of over or under dosage. A falling off in work and functional ability and mental dullness indicate overdosage. Hyper-activity, eccentric behaviour, increased delusions and hallucinations indicate increased psychotic mental activity, which calls for an increased dosage. High doses are often used in the acute stages and these may be reduced according to the previous criteria. Extra-pyramidal disturbances are common accompaniments but accasionally are due to other causes and then will not disappear when the drug has been reduced. Various neurological disorders, including poliomyelitis, may be overlooked in these circumstances.

*Senile Patients*

Agitation, aimless restlessness, and depression are common in seniles. Thes patients are particularly vulnerable to these drugs. Parkinsonism is easily produce and hypotensive attacks and collapse may occur. For these reasons, the margin ( safety of most of the tranquillizers and anti-depressants is too narrow. Sparine an Melleril are safer than the stronger drugs and probably as effective. They should b used in doses just sufficient to calm agitation. Somnolence and mental vacanc indicate over dosage. For the restless bed patient hypotension is not so dangerous and other drugs including Librium can be tried.

It is very difficult to decide whether apparent depression in old persons is genuin or due to memory failure. When the former seems likely, E.C.T. should be given firs followed by a mild tranquillizer or thymoleptic.

*Other Applications*

Insomnia in a tense anxious individual can be treated with single doses of Librium Muscular pain due to rheumatism, arthritis, or a fracture may be relieved by Libriu or other drugs which reduce muscular spasm. In anorexia or vomiting many of th drugs are used because of their anti-emetic effect but intestinal obstruction and reten tion have followed their use. At least one patient has been reported whose distentio and grossly uncomfortable flatulence disappeared when a tranquillizer that had bee prescribed to ease it was discontinued. Marsilid used in the treatment of angina an angina-like pain may produce euphoria and therefore mask genuine signs of dange Before prescribing it, it is wise to enquire whether the attacks of pain are related t emotional disturbances or not.

### SIDE EFFECTS AND COMPLICATIONS

Side effects are produced by practically all the tranquillizing and allied drug in particular those with the most active therapeutic effect. Patients vary in respons Schizophrenics are much the most tolerant. Side effects are particularly dangerous i the aged.

Giddiness, postural blacking-out, and collapse may be due to a fall in blood pressur caused by any of the drugs and can be serious in the aged and with cardio-vascula disease. Younger patients usually develop tolerance quickly and it is desirable, althoug not always possible, to get the patient to bed for the first three days of treatment.

Extra-pyramidal Parkinsonism is frequently seen and in most cases calls for reduce dosage. It can be controlled by Artane and similar drugs, where, as in schizophreni high dosage may be necessary, but it should not be allowed to develop in depressio or neurosis and is highly dangerous in the elderly.

Dry mouth is a common and unavoidable side effect frequently encountered wit Tofranil and it may even interfere with swallowing. Nausea, indigestion and restless ness are often complained of, especially with Stelazine. The insomnia of Parstelin ca usually be avoided by giving the last dose in the early afternoon.

Precautions are necessary to avoid contact dermatitis in nurses dispensing Chlor promazine and some others. Jaundice has been reported with many of the drugs especially the M.O.I. group and the Thymoleptics, with the exception of Parnate When jaundice or rashes appear the drug should be immediately withheld.

So far addiction has not been reported but there is some evidence that dependency if not addiction, may develop with Meprobamate, Parstelin, and the euphorisin drugs.

Tranquillizers, possibly because they do not convey the idea of sleep, have no replaced the sedatives and hypnotics as instruments of suicide, but their highly coloured capsules have an attraction for children and parents may not realize that they are dangerous.

### SOME PRECAUTIONS

Unless the doctor is sure that the patient understands and can be trusted, a responsible person should take charge of the drugs and of their administration. This is particularly important in most cases of depression and in schizophrenia.

Patients are most ignorant about tranquillizers and think they can take them if they feel strung up, and stop them at will. The doctor should explain the purpose of the treatment, insist on reliability and ask for signs of improvement, overdosage, or side effects to be reported. He should make it quite clear that the tablets are not to give temporary relief but to treat an illness. Some patients are scared of any drugs and will report headaches and sickness after each dose. In order to decide whether these are genuine side effects or not, the prescription can be changed to something innocuous such as Vitamin C; if the effects continue they are psychological; if not, probably toxic. Patients should be warned about the side effects they may expect.

The degree of good functional ability is the yardstick of improvement in depression and schizophrenia. Dullness, lethargy, and aimless inactivity, however, are more often due to over than under dosage. This can be tested by asking the patient to write a letter or make a little drawing. If there is overdosage the patient may have nothing to say and nothing to draw.

Phenothiazines should not be used in the treatment of severe and especially endogenous depression. However, small doses, preferably of the milder preparations, can be prescribed to control states of agitation in depression of various kinds.

Most of the thymoleptic drugs may cause Parkinsonism. Unless this is recognized the patient may be treated with heavier doses in the mistaken belief that the depression has deepened. An expressionless face, in which the furrows of depression are smoothed out, indicates Parkinsonism unless it is accompanied by increased motor activity. Parkinsonism is confirmed if the gait becomes slow and stiff or if tremors of the hands develop. The picture of Parkinsonism may not be very pronounced but it can always be suspected if the anti-depressant drugs seem to be producing a slowing of mental processes rather than acceleration and a blotting out of signs of agitation and depression.

In anxiety neurosis the initial dose should be small and the patient always warned of possible vasomotor disturbances.

Many of these drugs potentiate the action of other drugs and alcohol, as can be seen from the table, and it is wise to assume that all may have this effect. Therefore patients should be advised not to drive a car until tolerance to the drug has been established and they should not drive as long as there is any tendency to postural giddiness.

These drugs should not be prescribed for prolonged periods as is sometimes the practice with barbiturates. This is particularly dangerous in depression because patients incompletely cured and with the complication of drug side-effects are not infrequently seen, and there seems no justification for continuing to prescribe, for example, Tofranil, for twelve months in a cured or chronic depressive.

So far, legal action does not seem to have followed accidents and disabilities due to Parkinsonism and collapse, but it is wise to warn relatives and the patient that disagreeable complications may have to be expected.

### A REVIEW OF SOME OF THE TRANQUILLIZERS AND THYMOLEPTICS

It may be helpful to collate information and opinions from personal experience of some of these drugs. The pharmacological names of these new drugs are mostly long, and to have used them would have been difficult for the general reader; for this reason the familiar trade names have been used throughout where there is only on commercial preparation.

(1) *Tranquillizers*

*Largactil* (75—1,200 mgm daily). It is extensively used in the treatment of schizo phrenia and agitation. It may produce Parkinsonism, hypotension, liver damage an contact dermatitis.

*Stelazine* (2—30 mgm daily). A very valuable drug. Five times as potent as Largac til per mgm, it has the same indications and side effects.

*Majeptil* (15—300 mgm daily). A powerful tranquillizer and anti-emitic. Usefu in mania. It is not very suitable for outpatient treatment. Cases of intestinal obstruc tion and neurological disorder have been reported.

*Melleril* (75—600 mgm daily). An excellent mild tranquillizer for the treatment o anxiety and tension. It is claimed to be without any serious side effects.

*Sparine* (75—600 mgm daily). Another mild tranquillizer, particularly useful i geriatrics. Produces no liver damage.

*Modetin* (1—2 mgm daily). Somewhat similar to Sparine but more powerful. It i useful for a single daily dose. It is contra-indicated in depression.

*Serenace* (0·75—6 mgm daily). Probably not suitable for outpatient treatment bu useful in manic and schizophrenic excitement.

*Librium* (10—100 mgm daily). Relieves tension, insomnia, painful muscle spasn and states of anxiety and aggression. It is claimed not to produce Parkinsonism o autonomic blocking. A 10 or 20 mgm dose at night is given for insomnia.

*Taractan* (45—300 mgm daily). A rapidly acting drug. Contra-indicated wit alcohol and narcotics. It is a useful anti-depressant and for calming agitation.

(2) *Thymoleptics*

*Tofranil* (50—250 mgm daily). A powerful anti-depressant. It is apt to produc hypotension, Parkinsonism, and particularly dry mouth.

*Tryptizol* (50—250 mgm daily). Particularly suitable for endogenous depressio Has few side effects.

*Nardil* (30—75 mgm daily). A popular ant-depressant.

*Parnate* (10—30 mgm daily). May cause insomnia. It is claimed to be rapidl effective in depression and does not seem to cause liver damage.

*Parstelin* (Mixture—Parnate 10 mgm Stelazine 1 mgm). Has been tried in Barro Hospital in more than 300 cases of which about 100 were treated before it came on th market. It has proved to be a most useful drug in the treatment of difficult cases o anxiety-depression. It is usually given twice a day and need not be increased abo four doses maximum, though two daily are usually sufficient. The second dose shoul not be given after 3.0 or 4.0 p.m. as it may cause insomnia. It has been successful i terminating mild attacks of endogenous depression, as a maintenance treatment afte E.C.T., and to reduce anxiety attacks as a single dose before stressful occasions. Sid effects are few but collapse and brachycardia has been seen occasionally in old peop and it is probably better to avoid its use in old age. The restlessness sometime complained of may be due to the Stelazine in the mixture.

Largactil, Stelazine, Sparine, Serenace, Tofranil and Librium can be given b injection. Many of the preparations are obtainable in elixir form suitable for confuse or elderly patients.

There is of course an immense literature about these drugs which has been well edited and distributed by the drug firms. Many of the reports refer to treatment carried out in mental hospital on patients and in conditions very different from those met with in general and outpatient practice. This may account for some of the disappointments and contradictions experienced in using these undoubtedly valuable preparations. The ideal drug would be one which would have no side effects and precise therapeutic action and in which there would not be a very wide range of dosage. Above all, the physician should be able to make a clear assessment of progress without the obscurity of drug reactions. This perfect drug has not yet been evolved for any psychiatric condition.

